# Rapid Characterisation of Vegetation Structure to Predict Refugia and Climate Change Impacts across a Global Biodiversity Hotspot

**DOI:** 10.1371/journal.pone.0082778

**Published:** 2014-01-08

**Authors:** Antonius G. T. Schut, Grant W. Wardell-Johnson, Colin J. Yates, Gunnar Keppel, Ireneusz Baran, Steven E. Franklin, Stephen D. Hopper, Kimberley P. Van Niel, Ladislav Mucina, Margaret Byrne

**Affiliations:** 1 Department of Spatial Sciences, Curtin University, Bentley, Western Australia, Australia; 2 Curtin Institute for Biodiversity and Climate, Curtin University, Bentley, Western Australia, Australia; 3 Science Division, Department of Parks and Wildlife, Bentley, Western Australia, Australia; 4 School of Natural and Built Environments and Barbara Hardy Institute, University of South Australia, Adelaide, South Australia, Australia; 5 AAM Pty Limited, Perth, Western Australia, Australia; 6 Trent University, Peterborough, Ontario, Canada; 7 Centre of Excellence in Natural Resource Management, The University of Western Australia, Albany, Western Australia, Australia; 8 School of Earth and Environment, The University of Western Australia, Crawley, Western Australia, Australia; 9 School of Plant Biology, The University of Western Australia, Crawley, Western Australia, Australia; University of Rome ‘La Sapienza’, Italy

## Abstract

Identification of refugia is an increasingly important adaptation strategy in conservation planning under rapid anthropogenic climate change. Granite outcrops (GOs) provide extraordinary diversity, including a wide range of taxa, vegetation types and habitats in the Southwest Australian Floristic Region (SWAFR). However, poor characterization of GOs limits the capacity of conservation planning for refugia under climate change. A novel means for the rapid identification of potential refugia is presented, based on the assessment of local-scale environment and vegetation structure in a wider region. This approach was tested on GOs across the SWAFR. Airborne discrete return Light Detection And Ranging (LiDAR) data and Red Green and Blue (RGB) imagery were acquired. Vertical vegetation profiles were used to derive 54 structural classes. Structural vegetation types were described in three areas for supervised classification of a further 13 GOs across the region. Habitat descriptions based on 494 vegetation plots on and around these GOs were used to quantify relationships between environmental variables, ground cover and canopy height. The vegetation surrounding GOs is strongly related to structural vegetation types (Kappa = 0.8) and to its spatial context. Water gaining sites around GOs are characterized by taller and denser vegetation in all areas. The strong relationship between rainfall, soil-depth, and vegetation structure (R^2^ of 0.8–0.9) allowed comparisons of vegetation structure between current and future climate. Significant shifts in vegetation structural types were predicted and mapped for future climates. Water gaining areas below granite outcrops were identified as important putative refugia. A reduction in rainfall may be offset by the occurrence of deeper soil elsewhere on the outcrop. However, climate change interactions with fire and water table declines may render our conclusions conservative. The LiDAR-based mapping approach presented enables the integration of site-based biotic assessment with structural vegetation types for the rapid delineation and prioritization of key refugia.

## Introduction

Considerable changes in the distribution and ecology of species and ecosystems are likely to be ongoing over the coming decades in response to anthropogenic climate change [1]–[3]. Identifying refugia (habitats that facilitate species persistence during large-scale and long-term climatic change [4]), is increasingly important in conservation planning as a critical climate change adaptation strategy. Persisting in refugia may provide an important means of *in-situ* survival for many species [5]–[7].

Identifying the location of refugia requires a spatially explicit understanding of the relationships between biodiversity and the environment (including climate) at appropriate scales and through time. The current reliance on species distribution models (SDMs) is most often applied at coarse spatial scales, but refugia may occur at relatively fine spatial scales [8]–[10]. For example, the globally significant South-West Australian Floristic Region (sensu [11]; henceforth SWAFR) is predicted to experience a decrease in precipitation (e.g. [12], [13]), and coarse SDMs predict large impacts on species distributions [14], [15]. However, none of these models take into account fine scale environmental heterogeneity, and as a consequence are unable to identify refugia at finer scales - the scales likely to enable local persistence under predicted changes, though see [16], [17].

Emerging technologies such as LiDAR (Light Detection and Ranging) and RADAR (Radio Detection and Ranging) systems are powerful tools for the spatially explicit modelling of environment and biodiversity. The increasing availability of these tools enables ready mapping of vegetation structure including overstorey and understorey characteristics [18]–[20]. Delineation of spatial patterns based on structural characteristics can be related to vegetation types on the ground [21], [22]. Characterisation of vegetation structure further allows extraction of the key vegetation attributes of height and crown density [23], and can be used to quantify structural heterogeneity at local scales, and to identify environmental constraints and specific habitats. However, to identify refugia under projected climate change, vegetation structural characteristics (as measured by LiDAR or other remote sensing approaches) must be linked to predictive environmental variables. It may then be feasible to link structural vegetation mapping with local process-based measures to identify key vegetation types and places that are representative of refugia at finer spatial scales.

Ongoing changes and those projected under anthropogenic climate change are particularly important for mediterranean-climate ecosystems [24], [25]. These mediterranean-climate ecosystem regions occur on six continents [26], harbour a substantial proportion of the Earth's vascular plant flora [27], and are all recognized as global biodiversity hotspots [28]. The SWAFR is the least topographically complex of the five mediterranean-climate ecosystem regions with little opportunity for contraction to mountain refugia as the climate warms [26]. It is also predicted to be the most adversely affected by projected climate change, with consensus among global climate models that rainfall will continue to decline [13], [25], [29].

The SWAFR is characterised by the ancient granite-based landscapes of the Yilgarn Craton and Albany Fraser Orogen [30]. Granite inselbergs or outcrops (GOs) are topographically complex in comparison with the subdued surrounding landscape. Hence microclimatic variation, due to topographic and indirect effects of soil moisture variability [17], within GOs could buffer against regional climate change, and could continue to provide habitats for species occurring on or around them. Granite outcrops are generally rich in biodiversity, and are therefore of great conservation importance [31], [32]. In south-western Australia, at least 1200 vascular plant taxa are found on GOs [31], and GOs harbour a considerable proportion of the region's invertebrate, reptile, bird and mammal faunas [33], [34]. The importance of GOs is even greater in disturbed agricultural landscapes, where they constitute important habitat remnants for the biota [35], [36], and gene pools for surrounding landscapes under restoration.

The elevated nature and geological constitution of GOs means that they channel water, nutrients and plant residues to the fringes of the rock [37], [38], where growing conditions are more favourable for plants [39], [40]. This capacity may be important in south-western Australia, where moisture deficits, nutrient impoverishment, and acidity are typical features of local soils [41]–[43]. Weathering on exposed GOs provides nutrients and sediments to associated colluvial and alluvial fans surrounding the outcrops [40], reducing local constraints on plant growth. In addition, the slope and shallow soils of GOs reduce waterlogging, and basement rock beneath the fringe prevents water seeping away into deeper aquifers. Therefore, it is predicted that the fringes of GOs should have denser vegetation, greater biomass and higher productivity than the surrounding landscape [39].

Spatially explicit modelling of vegetation structure in conjunction with environmental variables will allow investigation of the interactions between vegetation characteristics and climate. A consistent characterization of local vegetation structure within a regional context should enable the quantification of habitats [44], [45] and the identification of environmental constraints influencing growth. Such an approach is based on the strong relationships between environment, vegetation type, and density of vegetation [46]–[48]. The five mediterranean-climate regions are commonly cited examples of convergent evolution in vegetation structure and function [27], [49]. Hence, some consistency in vegetation structure could be expected in similar environments across the SWAFR region despite remarkable floristic diversity (see for example [50]). Canopy height has been widely used to assess habitat condition and conservation status [21]. While correlations between canopy height/cover and environmental attributes are well known, there has been no previous attempt to provide fine-scale spatial realization of those relationships.

In this paper we establish relationships between vegetation structure and environmental variables to identify refugia using a case study of GOs across the SWAFR. We sought to generate detailed maps of local structural vegetation type as a means to relate growth to environmental variables indicative of local resource availability and growth constraints in topographically complex areas. This would need to be at a scale relevant to conservation management and the identification of refugia as safe havens for biodiversity. We have four specific aims and associated hypotheses.

1. Delineate and map local-scale vegetation structure across the region. We expect a wide variety of consistent vegetation structural forms applicable across the region reflecting variation in resource availability, despite local variation in species composition.
2. Compare the local spatial distribution of the types of vegetation structure near GOs across the rainfall gradient. We expect the tallest and densest vegetation to be confined to run-on areas at the base of GOs, because vegetation in these run-on areas have access to additional nutrients and water from the GO when compared to the surrounding areas.
3. Quantify the relationship between environmental variables and habitat attributes derived from vegetation structure. We expect a reduction in canopy height and cover of vegetation with reduced rainfall and soil depth.
4. Portray the vegetation structure predicted for sites under climate change projections for the region. We expect sites closest to GOs to retain proportionally denser and taller vegetation than sites further away from them. We also expect areas of tallest vegetation to be most affected by climate change.

## Materials and Methods

**Figure 1:**
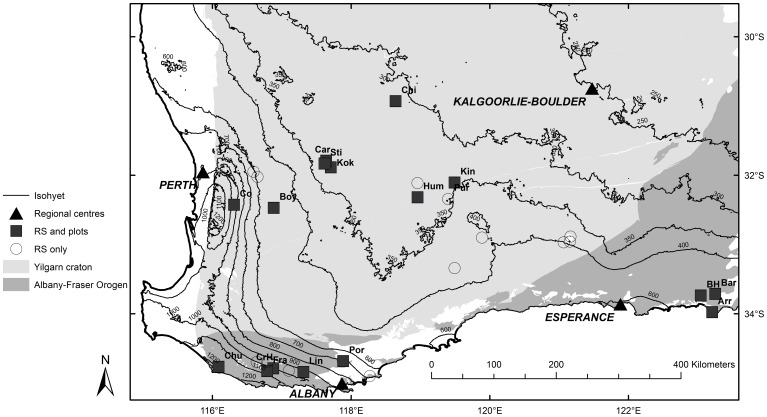
The location of 28 granite outcrops (squares) scanned using LiDAR and RGB imagery across the SWAFR climatic gradient. Detailed plot-based floristic surveys were carried out at 16 of these sites (filled squares with abbreviated names, see also Table 1). Isohyets are also shown. Closed triangles are regional centres.

Airborne LiDAR data and Red, Green and Blue (RGB) imagery were acquired by AAM Pty Ltd (Perth, Australia) from flights covering the areas around 28 targeted GOs across the SWAFR rainfall-gradient from mesic to low rainfall environments (Fig. 1). The selection of GOs was based on the inclusion of large and iconic GOs, while covering the full rainfall gradient with a limited number of flights, opportunistically including additional GOs in the flight path. The area bounded by the polygon connecting the surveyed sites was 296,361 km^2^. Within this region, LiDAR and RGB imagery were obtained over a total area of 95,485 ha.

### Acquisition of airborne LIDAR data with RGB imagery

Between 27 February and 2 March 2010 an airplane was flown over 23 GOs carrying an Optech 3100 LiDAR system and an Applanix DSS camera including RGB spectral bands covering 400–500, 500–600 and 600–700 nm, respectively. In April 2011, data were collected for the remaining five outcrops (Mount Chudalup, Mount Frankland, Mount Lindesay, Porongurups, Boyagin Rock) by airplane carrying a Leica ALS 50-II scanner with a Digital Mapping Camera from Z/I Imaging including RGB spectral bands covering 425–515, 515–590 and 600–650 nm, respectively. At both acquisitions, aircrafts flew about 1700–2200 m above the ground, and scanned approximately 1.5–2 km wide swaths, resulting in a distance between points on the ground of about 1.2 m and 0.63 points per m^2^, with a relative horizontal and vertical accuracy better than 0.24–0.35 m and 0.15 m, respectively. Both LiDAR systems recorded 4 discrete returns, with a footprint of about 0.39 m^2^. The RGB images were based on a ground sampling distance of about 0.2 m.

### LiDAR data processing

A description of the LIDAR processing is provided more fully elsewhere [22]. Overlapping areas of adjacent runs produced strips with a denser point cloud, these variations in point density being undesired [51]. Hence all layers were initially processed in a 4×4 m raster and, in each cell with LiDAR returns from overlapping runs only points were included from the run with the smallest mean scan angle. This produced grid cells with up to 32 returns per cell, although 11–15 returns were typically found in vegetated areas with trees.

A 2×2 m digital elevation model (DEM) of the terrain was derived from triangulation of all ground points, which was consequently used to determine the height above ground for all returns classified as non-ground (see [51] for a description of the procedure). Similarly, a 2×2 m canopy height layer was determined by subtracting the elevation of the ground from the elevation of each return. The DEM and selected layers of the GOs are downloadable (http://refugia.curtin.edu.au/). Other data-layers can be made available upon email request.

### Volumetric pixels

Subtle variations in point density occurred throughout the flight. Therefore, presence and absence of vegetation was recorded in 3D volumetric pixels (voxels) as these were less sensitive to these point cloud density variations than calculated percentages of returns per defined vertical layer. For each vertical layer of 1 m height, percentages of filled voxels (PVF) were computed within a 3×3 m window, producing a vertical PVF profile for each grid cell. These PVF profiles were smoothed in the vertical direction to better define the top and bottom of canopy layers by applying a simple Gaussian filter (with weights of 0.27, 0.46 and 0.27). Following the approach of Reitberger [52], a threshold of 20% (i.e. at least 2 out of 9 voxels) was initially used to trigger the start and end of a vegetation layer within a PVF profile. For the first two of these vegetation layers, canopy height, layer thickness, mean coverage and mean intensity were recorded. Mean intensity was based on only first and single returns, as return number has a profound influence on the recorded intensity [53]. The smoothed PVF profiles were further sampled at 18 height intervals from 1 to 80 m with intervals ranging from 1 to 20 m, increasing with height. These were stored for further processing.

### Identification of PVF profile classes

Local spatial heterogeneity in PVF profiles may identify patchiness that is significant for understorey and midstorey canopy layers. A hybrid classification procedure was used, aimed at using this spatial heterogeneity to identify vegetation-structural types. The sampled PVF profiles and canopy heights, cover, intensity and layer height of the top canopy layer (i.e. crown thickness) were transformed into principle components using standard image processing software (ERDAS ER Mapper®, Intergraph, Alabama, USA). Significant principal components, capturing 99% of the variation were used to identify unique PVF profiles for Mt Frankland National Park, and for Boyagin and Chiddarcooping Nature Reserves. These sites represented mesic, intermediate and dry environments, respectively. The iterative, self-organising (ISO, also known as the migrating means) clustering technique [54], was used to identify significant classes covering at least 1% of the area using ERDAS ER Mapper® software. For each of these classes, based on all pixels within these classes, minimum, maximum and median values per vegetation layer were determined. Also, typical vertical profiles were constructed and used to derive a qualitative description for each class. Profiles of the three areas were combined and similar profiles were removed, resulting in 54 unique PVF profile classes.

LiDAR data were further classified according to the type and elevation of the returns [44], where four vegetation layers were identified: low (<1 m above the ground), medium (1–3 m), high (3–10 m) and top canopy (>10 m) vegetation layers. The PVF profile classes were used in a pixel-based supervised classification using a minimum distance classifier for all areas. LiDAR intensity was excluded from the classification as the LiDAR intensity corrections for scan angle and flying height did not fully correct intensity differences between scanned areas. The minimum distance classifier is non-parametric, simple and fast and does not require dispersion statistics that need to be derived from training data [54].

### Conversion of PVF profile classes into structural vegetation classes

At selected locations in Mt Frankland, Boyagin and Chiddarcooping, vegetation types characterised by a similar floristic composition and vegetation structure were described and the presence of dominant overstorey and understorey species was recorded. Field based geocoded photographs were taken at the same locations. These locations were selected on transects covering a wide range of PVF profile classes to ensure that visited locations were representative of variation in structural vegetation types. Variations in the PVF profiles within an area with similar vegetation typically represented a range of understorey conditions and disturbance history. Typical combinations of 54 PVF profile classes were identified (based on similarity in vertical profiles and co-occurrence within a single vegetation type after examination of RGB imagery), and merged into 27 meaningful broader structural vegetation classes. Thus each of these structural vegetation classes were related to at least one of the vegetation types observed.

A segmentation step was used to derive so-called objects or polygons based on canopy height at local (i.e. a large tree) and slightly larger “vegetation” scale by adjusting the scale parameter (eCognition Developer® 8, Trimble Geospatial Imaging, München, Germany). These polygons were classified according to the structural vegetation class with the largest relative coverage. Bare areas without above-ground LiDAR returns within native vegetation areas were further subdivided into smaller polygons based on RGB brightness (i.e. the sum of the digital numbers in all three bands). Thresholds based on local brightness differences could be used to differentiate bare rock from moss-mats for those outcrops in higher rainfall areas where moss-mats are darker in colour. Thus, the brightness, derived from the RGB values, within these smaller scale polygons was compared to the brightness of vegetation scale polygons. They were assigned to the moss-mat class if between particular thresholds, with thresholds iteratively adjusted for each outcrop. For dryer areas, moss mats were less prominent and also less visible in summer when the aircraft was flown. There was also considerable variation in illumination across any individual outcrop. Therefore brightness of moss-mats either did not contrast strongly, or differences were small compared to variation in brightness across particular outcrops. Consequently, an approach based on local brightness differences could not be used, and moss-mats were not further classified for these drier areas.

### Supervised classification

Delineations based on structural characteristics are most valuable when they also identify transitions between vegetation types. Structural vegetation classes can effectively be assigned to vegetation types when combined with local expert knowledge [22]. However, within a regional context, structural vegetation classes are not specific for a vegetation type. Therefore field records for validation were collected in transects at Boyagin (135 records), Chiddarcooping (25 records), Porongurups (106 records) and Mt Frankland (168 records) to determine whether identified structural vegetation classes were related to floristic vegetation types, or to transitions between them. The observations included locations with various periods of recovery after fire. Kappa coefficients, indicating classification accuracy [54], were determined for Boyagin, Porongurups and Mt Frankland.

Means were determined for canopy height and for ground cover for each structural vegetation class, based on all classified polygons within Boyagin, Mt Frankland and Chiddarcooping.

### Floristic surveys and plots

A total of 16 GOs were selected for detailed study (Fig. 1). Between 25 and 36 plots (see below) were established for most GOs with the exception of Boyagin Rock where 71 plots were established. At each of these 16 GOs, plots were divided between three major habitat types: 1) sites either with shallow soils with water off-flow or seasonally inundated soil-filled rock-pools, also known as gnammas (OF, herbfield vegetation); 2) sites with moderately deep soils or with vegetation with access to cracks in the rock with on- and off-flow of water (INT, usually with shrubs or low trees); and 3) on-flow areas at the base of the outcrops (ON, typically with forest vegetation – particularly in higher rainfall areas).

Sizes of plots were based on accumulation curves for species and the size of taxa under investigation (i.e., 1×1 m for OF plots, 5×5 m for INT plots and 20×20 m for ON plots). Plot locations were recorded with a hand-held GPS. Floristic composition (all vascular plants) and soil depth was recorded for each plot, using a soil depth probe hammered or pushed into the ground by hand until maximum depth (or at least 50 cm for deeper soils), at 5 random locations in the plot. These were used to calculate the probability of a soil deeper than 0.5 m (pDS). A total of 494 plot locations were available across the 16 sites with associated LiDAR data.

### Statistics for plot locations

Statistics concerning canopy height and ground coverage were calculated based on a 4 m buffer around the geocoded plot locations, intersected with classified polygons. The buffer was used to account for the error made when recording the position using a hand-held GPS. Means of ground coverage and maximum canopy height were determined for each plot (including buffer). In addition, weighted values were determined from structural vegetation class means, based on all intersected polygons that cover at least a quarter of a plot.

The influence of environmental variables on canopy height and ground cover was tested with a simple linear regression model. Multivariate linear models (Y = αX+e) were fitted with interactions included. P-values were evaluated for each variable in the explanatory X-block and variables were excluded when not significant. Potential explanatory variables included annual precipitation (interpolated values from the WorldClim dataset based on means of the years 1950–2000 [55]), elevation range (difference in elevation between highest and lowest elevation plots for each GO) and pDS. Elevation range may be a proxy for potential runoff and may also influence rainfall, particularly in coastal zones. Robustness of relationships was evaluated with a leave-one-out (LOO) validation, using the Q^2^ statistic [56].

### Refugial capacity

For the SWAFR, the A1F1 scenario, including high CO_2_ emissions predicts a greater than 40% chance of exceeding a 20% reduction in rainfall when compared to the 1961–1990 reference period for climate in 2070 [57]. We used this 20% reduction to illustrate potential changes in vegetation structure and the utility of the methodology derived.

The surveyed plots were located along the rainfall gradient from mesic in the High Rainfall Province (>600 mm rain p.a. – both Yilgarn Craton and Albany Fraser Orogen sites) to the inland side of the Transitional Rainfall (Yilgarn) and eastern edge of the Southeast Coastal (Albany Fraser) Provinces (both 300–600 mm rain p.a. [11]), with differences in vegetation structure reflecting these gradients. To enable use of these relationships for deriving projections around GOs, spatially explicit values for each polygon were needed for all terms included. For pDS, these values were estimated using regressions based on current vegetation structure and rainfall, using linear regressions based on mean values derived from the assigned structural vegetation class covering plot areas. Estimated pDS values, maximum supported canopy height and ground cover were determined for each polygon using the present mean vegetation height and ground coverage.

Under the assumption that current relationships between environmental variables and vegetation structure that vary along a spatial gradient can be used to predict an *in-situ* change over time, these relationships can be used to assess the impact of new climate regimes across the region. This means virtually “relocating” current outcrops to new climate regimes equivalent to current climates in lower rainfall areas. For each polygon, the current structural vegetation classification and means of environmental variables were exported (to a simple spreadsheet). The equations describing relationships between environmental variables and canopy height for plot locations, as described above, were used to predict future canopy height under an A1F1 scenario with a 20% rainfall reduction. This was repeated for ground cover. The distance between predicted canopy height and ground cover and mean values of each structural vegetation class were calculated and used to reclassify each polygon. Individual classes are heterogeneous and mean canopy height and ground coverage of a single polygon can be much higher than the mean of the class where the polygon was assigned to. This because many other features were also included in the supervised classification. Thus a small change in canopy height or ground cover may lead to a reclassification into a class with larger mean canopy height or ground coverage. Reclassification was therefore restricted and only vegetation structure classes with a lower or equal mean canopy height and ground cover than the class currently assigned to the polygon could be selected. From these, the vegetation structure class with the lowest minimal distance was selected and linked to the polygon for display.

## Results

The canopy height of vegetation in plots on or near GOs (see Fig. 1. for the locations of the GOs) responded more strongly to precipitation than to ground coverage, with annual precipitation ranging between 314–1208 mm yr^−1^ for the GOs included in this study (Table 1). For most GOs, mean and maximum canopy height was taller and ground coverage higher in on-flow plots than in intermediate or of-flow plots.

**Table 1:** Granite outcrop study areas, showing rainfall (mm yr^−1^, WorldClim dataset), elevation range (ER), mean canopy height and ground coverage for plots in off-flow areas (OF), intermediate sites with on- and off-flow (INT) or in on-flow areas near each outcrop (ON).

Study area	ER	Precip.	Canopy height (m)				Mean ground coverage (%)		
	(m)*	(mm)	OF	INT	ON	ON (max)	OF	INT	ON
Mt Chudalup (Chu)*	99	1208	2.05	2.63	28.33	33.38	13.74	40.30	92.79
Mt Frankland (Fra)	115	1044	3.10	5.29	31.10	40.43	16.34	44.71	87.75
Crossing Hill (CrH)	76	996	0.83	2.72	10.27	14.22	38.40	44.83	73.92
Mt Cooke (Co)	239	962	0.07	0.99	5.16	11.98	8.35	36.02	44.04
Mt Lindsay (Lin)	304	922	0.07	0.20	5.67	10.70	21.25	10.37	65.88
Porongurups (Por)	335	687	0.23	1.66	23.12	33.24	23.60	27.25	89.47
Cape Arid (Arr)	346	514	0.10	0.58	0.35	1.48	7.95	24.27	28.02
Boyagin Rock (Boy)	79	512	0.14	0.79	5.94	12.20	10.30	32.58	42.70
Boyatup Hill (BH)	46	488†	0.05	0.16	1.85	3.19	5.65	27.56	71.87
Mt Baring (Bar)	128	466†	0.10	0.16	1.15	2.37	12.60	17.78	51.89
King Rocks (Kin)	64	338	0.02	1.47	4.96	9.30	4.90	27.37	42.48
The Humps (Hum)	84	333	0.06	2.11	4.71	7.32	2.93	36.04	46.40
Mt Stirling (Sti)	77	332	0.57	1.11	6.78	10.71	12.70	25.98	47.61
Kokerbin Rock (Kok)	72	324	0.25	1.22	5.61	10.04	8.89	33.36	52.56
Mt Caroline (Car)	60	326	0.58	2.20	4.32	7.30	10.64	41.60	40.46
Chiddarcooping (Chi)	124	314	0.19	0.69	5.45	8.04	16.43	23.55	37.21

Based on extremes in the elevation of plot locations.

Rainfall appears high, but is probably reasonable, considering nearby Mt Howick rainfall of 379 mm (1994–2012, DAFWA) at approximately the same distance from the coast.

The abbreviation for GO in parenthesis is as in Figure 1.

### Structural vegetation types

**Figure 2:**
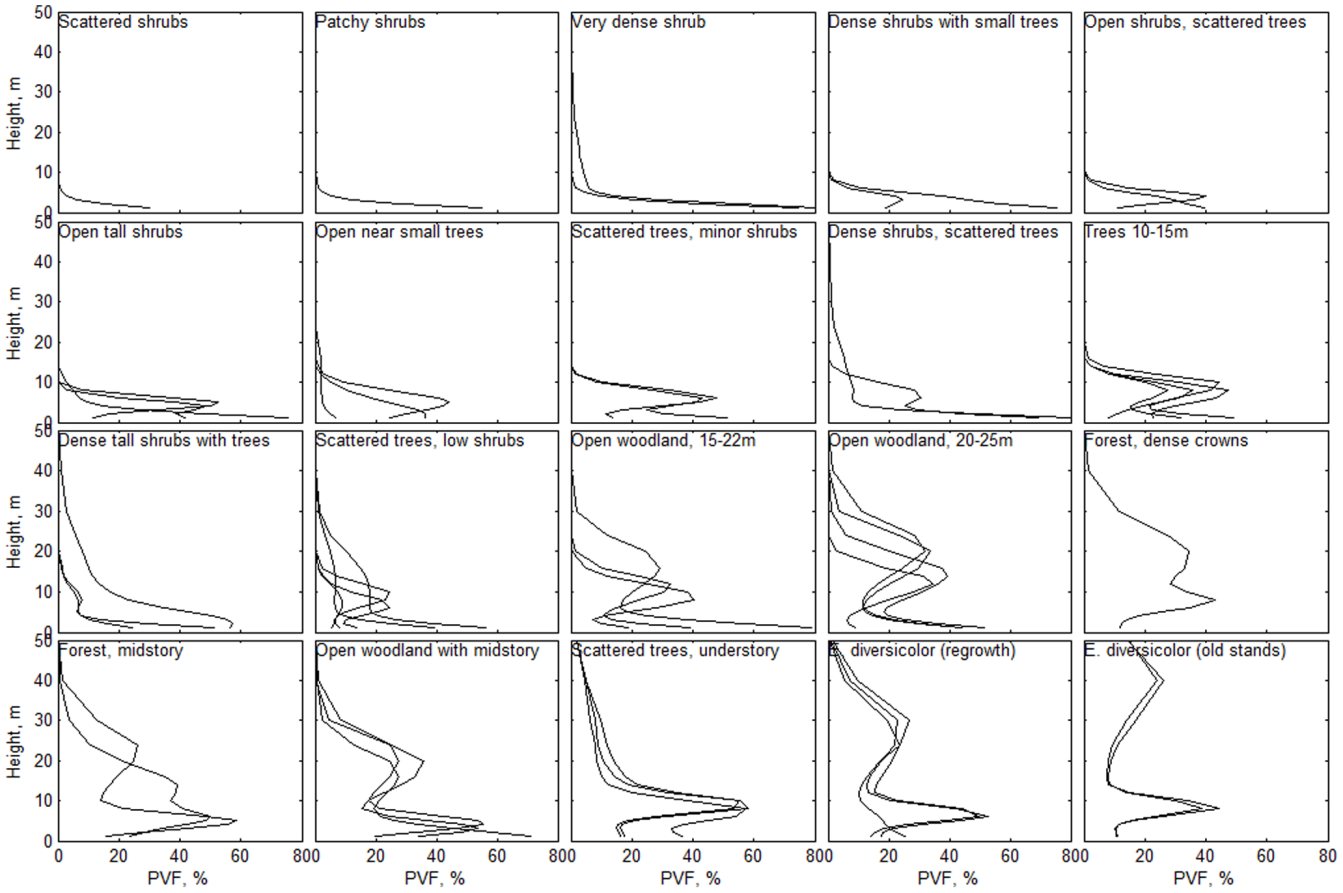
Vertical profiles showing the smoothed percentage of voxel fill (PVF) within a 12 by 12 m spatial window (3 by 3 pixels) as a function of height above the ground surface. Each line indicates a unique PVF, and multiple PVFs were assigned to a structural class if occurring within a ground-truthed vegetation type.

The vertical vegetation profiles generated showed a wide range in canopy height, distribution of canopy elements and ground cover across the region (Fig. 2), reflecting the range of structural vegetation types present. Although each individual PVF was distinct, groups of PVFs with similar vertical distribution profiles, but differences in ground cover, can be recognized. Some structural classes included a wider range of PVFs than others (e.g. the Open woodland class represents a wide range of vegetation types). However, these PVFs and structural classes can be linked to structural vegetation types when combined with topography and local expert knowledge [22]. In some areas, classes with a distinct vegetation structure can be directly linked to floristic vegetation types. For example, tall open-forest dominated by *Eucalyptus diversicolor* (Myrtaceae, karri) had a very distinct profile (Fig. 2), which in some areas can be further linked to the age of stands. Thus, in the area of Mt Frankland, substantial areas of karri regrowth show a distinct profile in comparison with old-growth stands dominated by the same species. However, in other situations, distinct profiles may be associated with multiple vegetation types, reflecting differences in landscape position and an array of species composition. For example, combinations of these profiles can be directly linked to vegetation types when combined with local descriptions for Mt Frankland and the Porongurups (Kappa coefficients of 0.86 and 0.78 respectively), and when combined with local expert knowledge for Boyagin, see [22].

### Comparative spatial distribution of vegetation types

**Figure 3:**
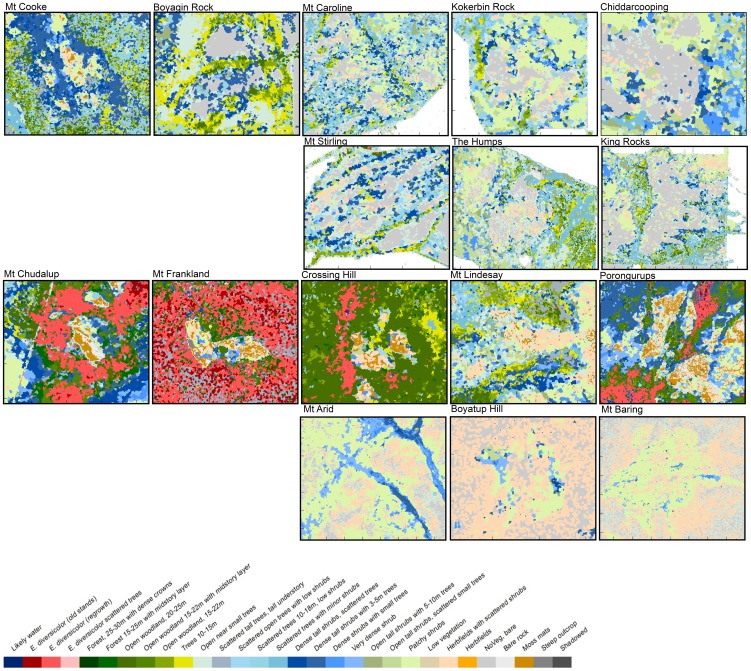
Comparison of vegetation structure on and around a granite outcrop in each of eight areas in the Yilgarn Craton (top) and the Albany-Fraser Orogen (bottom), indicatively displayed according to decreasing annual rainfall from left to right.

For all GOs, vegetation is taller and denser in on-flow areas at the base of the outcrops (Fig. 3). Tall karri trees (to 70 m in height) dominated on-flow plots with highest annual rainfall (i.e. Mt Chudalup and Mt Frankland), and at sites with relatively large topographic relief (i.e. the Porongurups which influences local climate). However, karri is restricted to the highest rainfall and least seasonal end of the High Rainfall Province. Hence vegetation height was much lower elsewhere, even in on-flow sites.

On GOs, low and open vegetation associated with shallow and rocky soils is dominant, usually covered with shrubs, herb fields or moss mats. Vegetation types with similar structure can be found across the rainfall gradient. Canopy height in off-flow areas for Mt Frankland and Mt Chudalup were taller than may have been expected. However, taller and denser vegetation occurs further from outcrops in higher rainfall parts of the Transitional Rainfall and Southeast Coastal Provinces but is confined to narrow fringes in on-flow areas near outcrops in lower rainfall areas.

In the High Rainfall Province, low and open vegetation in shallow soils occurs only on granite outcrops. However, this vegetation structure (within the study sites) is replaced by denser and taller vegetation where soil depth increases, in soil pockets on the GOs, and in areas surrounding them. In high rainfall and swampy areas, dense shrublands occur in lower landscape positions (e.g. surrounding Mount Chudalup and Mount Frankland). At the lower rainfall end of the Transitional Rainfall and Southeast Coastal Provinces, low, open vegetation with patchy or scattered shrubs is also common on deeper sandy soils with limited water holding capacity further from the outcrop.

### Environmental variables and habitat attributes

The average height and ground cover in the polygons overlapping the plot locations were related to environmental factors (rainfall, elevation range and soil depth). The R^2^ values of the simple regression models ranged from 0.48 to 0.91 for maximum canopy height, and from 0.39 to 0.84 for ground cover (Table 2). For canopy height in on-flow plots the elevation range is a significant term, and in combination with rainfall, reflects the importance of moisture regimes in these sites. Separate models run only with the inclusion of off-flow and intermediate plots had low explanatory power (R^2^<0.1, not shown).

**Table 2:** Linear regression and leave-one-out (LOO) validation statistics of multiple linear regressions with parameter estimates of environmental factors determining maximum canopy height and ground coverage.

Means	N	R^2^	LOO Q^2^	intercept	Rainfall	EV	pSD	EV×pSD	R×pSD	R×EV
	*Maximum canopy height*									
All plots	524	0.50	0.49	2.42	−0.00015*	−0.013‡	−3.718†	0.026‡	NS	NS
On-flow plots	144	0.48	0.44	2.78	0.008*	−0.107‡	6.512†	NS	NS	0.0001‡
Type/GO	48	0.65	0.55	1.54	−0.001*	NS	−4.271*	0.027‡	NS	NS
Classification	22	0.91	0.83	0.08	0.0074*	−0.051†	−5.932*	NS	0.040‡	NS
	*Mean canopy height*									
Classification	22	0.92	0.85	1.26	0.0034*	−0.057‡	−11.197*	NS	0.044‡	NS
	*Ground coverage*									
All plots	524	0.39	0.38	21.94	−0.001*	−0.056‡	5.189*	0.079‡	0.034‡	NS
On-flow plots	144	0.53	0.50	33.93	0.0197‡	NS	NS	NS	NS	NS
Type/GO	48	0.78	0.70	22.2	0.0004*	−0.068‡	5.661*	0.095†	0.035†	NS
Classification	22	0.84	0.78	−19.79	0.047‡	NS	64.755‡	NS	NS	NS

The explanatory variables included annual rainfall (R, mm), probability of a soil deeper than 0.5 m (pDS), elevation range (EV, m) and their interactions, and granitic substrate. Insignificant terms (p<0.05) were excluded from the fitted models. Models evaluated were 1) all individual plots, 2) only on-flow plots, 3) geometric means for each plot type per outcrop and 4) geometric means of structural class polygon attributes covering at least 20% of plot areas.

Not significant;

p<0.05;

p<0.01;

Transect and elevation range were.

**Figure 4:**
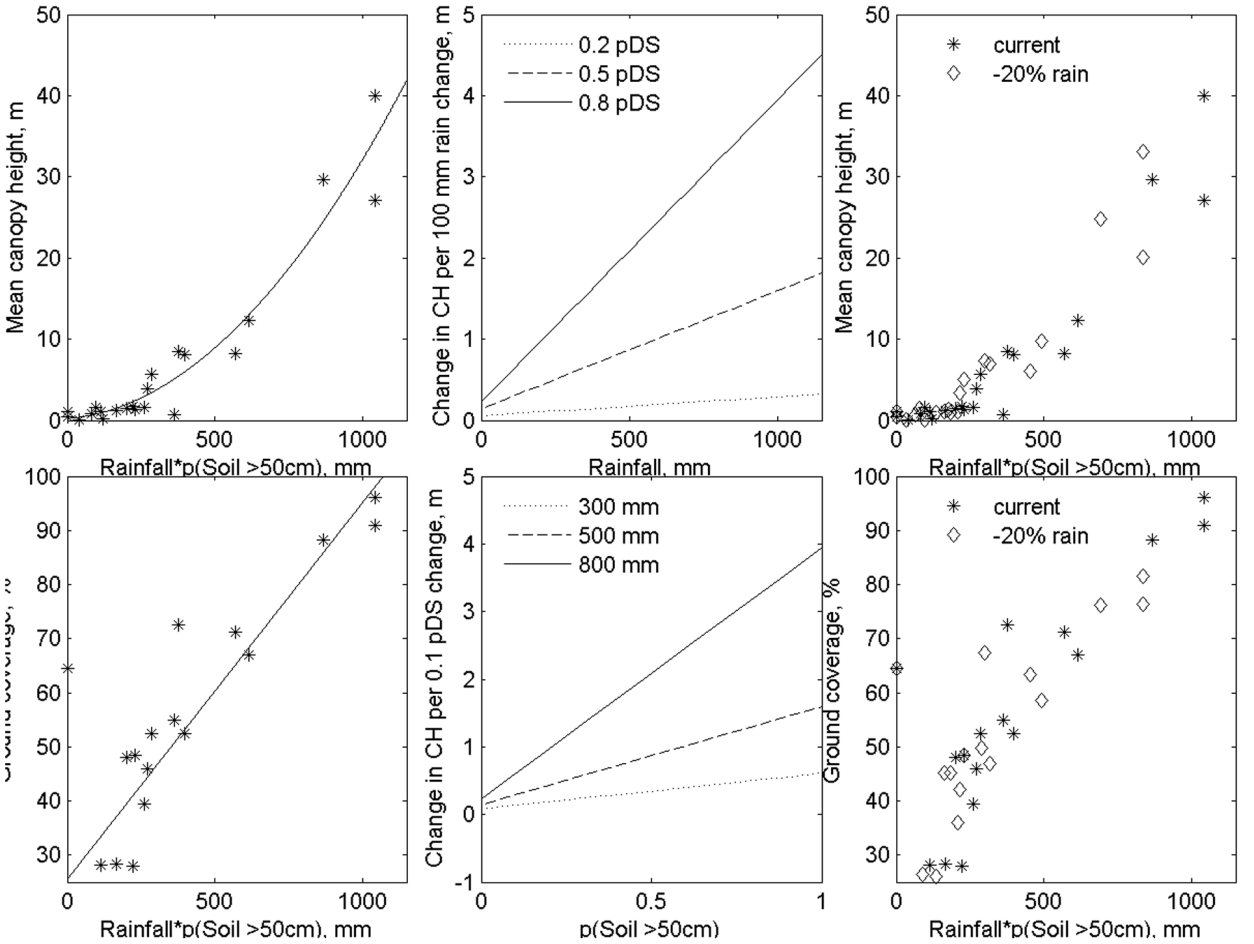
Combined effects of annual rainfall and the probability of a soil deeper than 0.5 m on mean canopy height and ground coverage in plots on 16 granite outcrops across the SWAFR. Each point indicates the mean value derived from all plots with the same structural class. Equations fitted (with x = pSD×R): Ground cover = 25.5+69.6E-3x (R^2^ = 0.75); Canopy height = 0.21+28.4E-4x+2.9E-5x^2^ (R^2^ = 0.93). The right-hand side figures indicate the current and future canopy height and ground cover for these class means.

When plots were grouped according to structural vegetation type, strong relations with environmental factors emerged (R^2^ value of 0.92 and 0.84). The models were also robust, with a large positive value for Q^2^ in the leave-one-out validation of the relationships. A combination of soil depth and annual rainfall resulted in relationships with R^2^ values of 0.75 for ground cover and 0.93 for canopy height (Fig. 4), with each point in this relationship representing a different structural class in plots on or near outcrops.

The absolute changes in canopy height and ground cover are larger per mm of rainfall reduction for patches with a larger pDS. With an equally strong change in ground cover for shallow soils, the influence on structural vegetation types may be much larger for shallow rocky areas. For example, in an area with 500 mm rainfall, a 100 mm reduction in rainfall on soils with a pDS of 0.5 results in a change in canopy height of about 1 m. This reduction in available water can be compensated by moving to a site with a pDS of 0.7 (see Fig. 4).

We found that the pDS can be modelled using attributes of the structural vegetation maps combined with rainfall (R) values: pDS = 0.55+(10.89×CH+8.93×GC−0.73×R)/1000. Canopy height (CH), ground cover (GC) derived from LiDAR and rainfall explained 85% of the variation in pDS (N = 22, R^2^ = 0.85, LOO Q^2^ values of 0.76). This relationship allows estimation of the pDS of the wider surroundings of GOs, as the vegetation of those areas will also reflect their environment. Some structural vegetation types represent a transition (e.g. karri regrowth represents an intermediate phase in the life of the forest stand) in vegetation structure.

### Refugial capacity

The means of canopy height and ground coverage within structural classes were directly related to environmental factors along the climate gradients in this study. Relationships were strong enough to meaningfully substitute space for time in assessing the impact of new climate regimes across the region.

**Figure 5:**
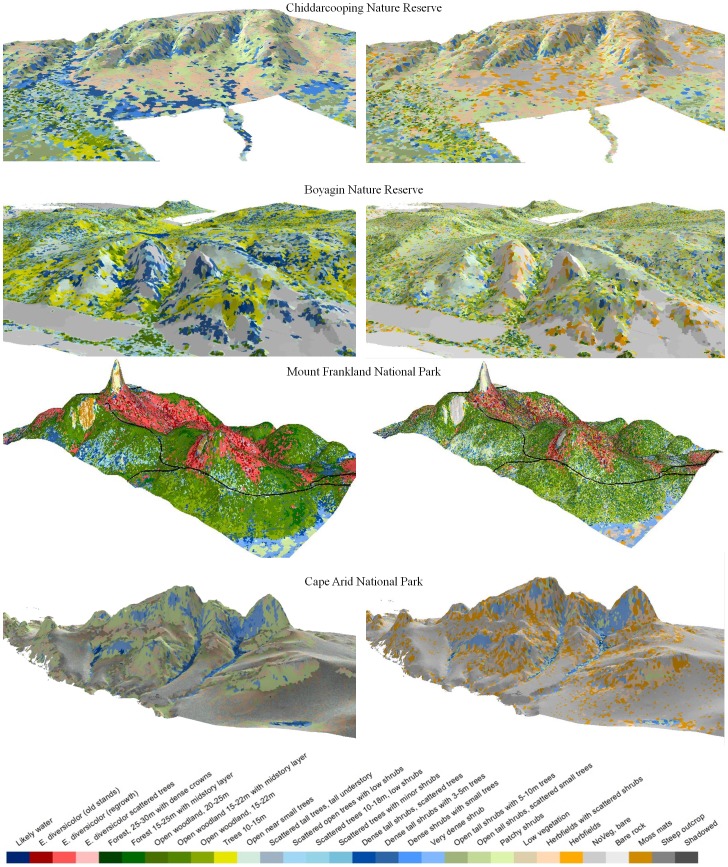
Diversity and spatial relationships with topography for current (left hand side) and future (2070) vegetation structure under a 20% rainfall reduction scenario (right hand side) on four granite outcrops (GOs) in the SWAFR. Areas surrounding GOs are shown from a birds-eye view with an elevation exaggeration of four.

**Figure 6:**
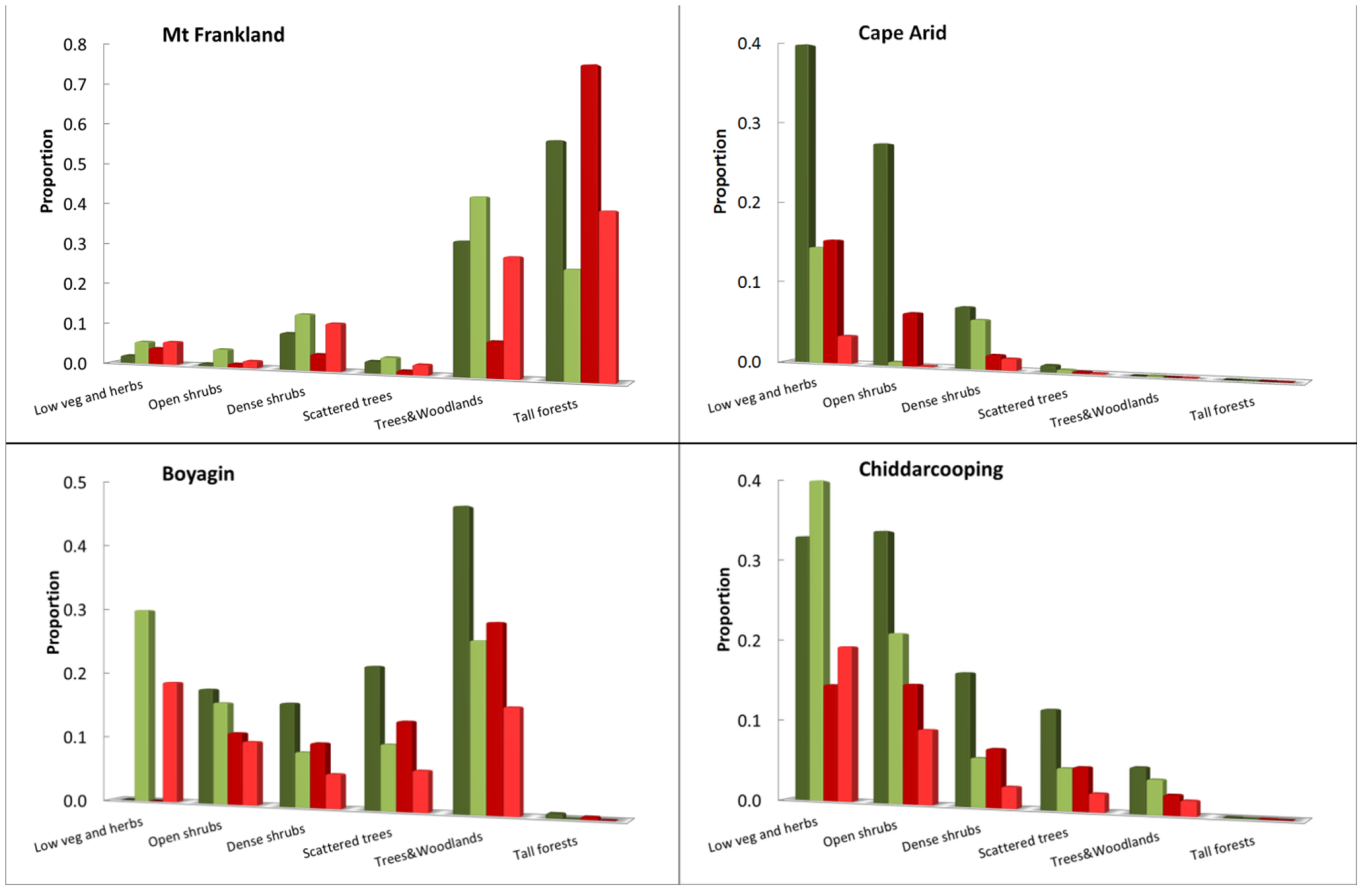
Current proportion (dark shade) and expected change in proportion (light shade) of the area covered by vegetation types under a reduced rainfall scenario for the four areas with the same extend as the areas displayed in Figure 5 (Green) and directly around granite outcrops within areas with the same extend as displayed in Figure 7 (Red).

There is a significant reduction in the area covered by vegetation with taller and denser canopies with a 20% reduction in rainfall (Fig. 5). Higher rainfall areas show a proportionally much greater change in both height and cover with this rainfall reduction (Fig. 6). For example, vegetation from Mt Frankland changes from Tall open-forest dominated by karri to scattered areas of karri forest. This structural vegetation type is projected to contract to water gaining areas, where currently the tallest trees are supported. For example, much of the current vegetation in the Open woodland 20–25 m class, typically including *E. guilfoylei* (yellow tingle) in association with *Corymbia calophyla* (Myrtaceae, marri) and *E. marginata* (jarrah), is projected to contract to narrow fringes surrounding the area of tall karri forest. Low, dense vegetation and shrubland found on Mt Frankland is projected to be replaced by a low and more open vegetation structure. However, dense shrub vegetation was not found on areas surrounding the rock in future climates, as taller vegetation was still supported on deeper soils. A strong reduction in proportional area for this habitat type is expected. However, in the wider landscape, the proportional reduction in dense shrub was much smaller (Fig. 6) than that of taller vegetation.

**Figure 7:**
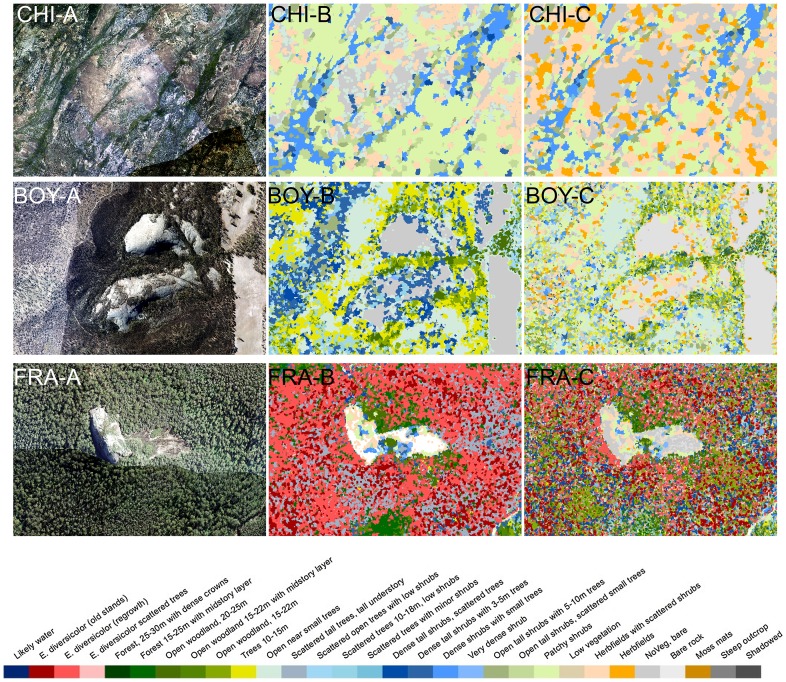
RGB images (A) and structural vegetation classes (B) of current vegetation and projected structural vegetation classes based on a 20% rainfall reduction scenario (C) zoomed to areas surrounding granite at the surface in Chiddarcooping (CHI), Boyagin (BOY) and Mount Frankland (FRA).

For Boyagin, the areas classified in the Trees 10–15 m class, with *Allocasuarina huegeliana* (Casuarinaceae, rock sheoak) and *Eucalyptus accedens* (Myrtaceae, powder-bark wandoo), are projected to be replaced by shrubs with scattered trees, a vegetation class now including kwongan vegetation [58]. The total proportion of this open shrubland vegetation is expected to decrease greatly (Fig. 6), and the shallow gravelly soils currently supporting kwongan vegetation will likely become low vegetation. There is a major projected reduction in patches with dense shrubland or scattered trees on Boyagin Rock (Fig. 6 and Fig. 7), with these structural vegetation types contracting to the base of the outcrop.

Areas with deeper soils along waterways below GOs are also important for relatively dense and tall vegetation. For example, in Chiddarcooping, *Eucalyptus salubris* (gimlet) or *E. salmonophloia* (salmon gum) occur in deeper soils, classified as Trees 10–15 m (Fig. 5). Areas further from streams will only support vegetation classified as Open tall shrubs or Scattered shrubs, reflecting a much more scattered and open vegetation, with likely changes in composition. The proportional changes in vegetation types in the wider landscape are expected to be more pronounced than those directly surrounding the outcrop (Fig. 6). The dense vegetation in narrow fringes around the base of GOs, typically including *Acacia lasiocalyx* (Fabaceae, rock acacia), and rock sheoak, is projected to further contract and become more open. On the GOs, Patchy or Scattered shrubs on shallow soils are likely to be replaced by low vegetation or herbfields with annual plants (Fig. 7).

## Discussion

We have provided a rapid characterisation of local-scale vegetation structure in relation to environment across the SWAFR. This has enabled the identification of fine-scale patterns of vegetation structure, and projections under anthropogenic climate change. A warmer, drier climate means that ecophysiological thresholds of some species may be reached locally in areas due to spatial variation in topography and radiation [9]. In this light, prudent management for conservation is likely to focus on areas such as refugia [59], where biodiversity may be able to persist for longest [4], although careful experimentation on ecophysiological thresholds is needed to test this hypothesis.

LiDAR-based mapping of vegetation structure can highlight specific areas for potential conservation and protection within a broader regional context. However, the assumption that spatial relationships between vegetation structure and environment can be used to predict *in-situ* temporal changes needs further critical testing. Our combination of spatially explicit mapping of structural vegetation types with environmental variables and site-based biotic assessment enabled the rapid delineation and prioritization of key potential refugia.

### Structural vegetation types

We found that LiDAR can be used to quantify differences in vegetation structure at a local scale [44], [53], [60], [61]. Canopy height in off-flow areas for GOs in very high rainfall areas was taller than expected due to the proximity of plots to tall vegetation, affecting the LiDAR derived height estimates. Tall vegetation strongly influences height in neighbouring raster cells due to the triangulation of top canopy returns, and some of these cells may have been included in the 4 m buffer that was used, affecting the height estimate of these herbfield plots.

The voxel-based characterization used here [22], allowed explicit characterization of vertical profiles for every pixel, despite low density point clouds. The identified classes reflected differences in canopy height, density and the vertical distribution of vegetation. The wide range of LiDAR instruments and techniques available for canopy characterization [20] provide the means to translate plot-based assessments to larger areas in a wide range of studies.

Typically, single vegetation types include several vertical profiles, mostly occurring in regular spatial sequences (e.g. a dense tree adjacent an open shrubland), and a single vertical profile may occur in several vegetation types [22]. Vegetation structure may be disturbed in fire-prone landscapes [62], [63]. However, the structural classes recognised here were predominantly based on the overstorey, with the understorey being of secondary importance. Hence, the accuracy of the overall classification was not strongly influenced by the presence of various recovery periods after low intensity fire as observed in the visited locations, although high intensity or frequent fire may have very different consequences.

The importance of water gaining on-flow areas at the base of granite outcrops has long been recognised [38], [64], [65]. We found that these areas support denser and higher vegetation when compared to the immediate surroundings. However, as expected, they also share structural similarities to the vegetation in higher rainfall areas.

### Environmental variables and habitat attributes

This study provides a methodology to link vegetation structure with environment at fine spatial scales over a broad geographic area. As expected, we found that rainfall and soil depth had a significant influence on vegetation height and ground cover in plots located on and around outcrops. Other environmental variables that were not considered in our study, e.g. the amount of water influx, and availability of cracks in the rock [66], may also be important.

The novelty of our approach is that it makes the relationships between environment and vegetation structure spatially explicit at a fine scale, and reveals potential associated patterns in relation to predicted climate change. For on-flow plots, significant interactions between elevation range and rainfall were found for canopy height, indicating the importance of runoff from the outcrop. However, elevation range was not significant when explaining differences in ground cover. The relationships based on values from individual plots were different between GOs in the High Rainfall Province and other areas. This difference was accounted for when averaged over structural class means, demonstrating that our approach can be applicable across the wider SWAFR.

The classification of vegetation structure at local scales enables a quantification of environmental drivers for important habitat characteristics such as vegetation height and cover. There was a direct relationship between rainfall, soil-depth and vegetation structure, suggesting that water availability is the major driver of vegetation structure in these environments. The strength of this relationship may be illustrated by the strong crown decline in response to the reduction in rainfall in recent years [67], [68], with a higher incidence of crown dieback on soils with stony profiles and low water holding capacity during the 2010/2011 summer which was the hottest and driest on record [69].

The vertical distribution of canopy elements is strongly correlated with the diversity of vascular plant species [70] and with faunal diversity [60]. A change in vertical structure due to a reduction in rainfall therefore has direct implications for biodiversity. Monitoring of vegetation structure with LiDAR provides a means of assessing overall habitat condition, and ecophysiological response to changing climate [70]. These strong relationships between vegetation structure and climate indicate that the structural vegetation map may also be used to identify environmental constraints within the regional context for areas directly around GOs. It should, however, be noted that further away from outcrops, other constraints, such as waterlogging or salinity, may be of greater importance than the proximity of the GO in determining vegetation height. Therefore, care is required when extrapolating the relationship between rainfall, soil depth and vegetation structure where the response to climate change may be very different.

### Comparative spatial distribution of vegetation types

The comparison of vegetation structure on plots across a rainfall gradient has provided a means of understanding spatial patterns within the landscape context that is an essential element of the identification of refugia [4]. Similar vegetation structure was found across the rainfall gradient, but their landscape position varied predictably in relation to water availability. Vegetation types occurring within the broader landscape in the mesic end of the gradient were confined to on-flow areas at the lower rainfall end. These on-flow areas have access to more water [38], and consequently may also have unique microclimates resulting from the topography and vegetation structure [10], [17], [71]. In more mesic areas, low and open vegetation is confined to outcrop areas, whereas these structural vegetation types were dominant on GOs in the lower rainfall areas of the region.

### Refugial capacity

Under projected climate change, reductions of up to 20% in rainfall in comparison with the base period 1960–1990 can be expected by 2070 [57], emphasising the importance of so called drought refugia [6]. This indicates that granite outcrops exhibiting a wider range of habitats and water gaining on-flow areas may facilitate species persistence – important characteristics of refugia under climate change.

A close connection between rainfall and catchment groundwater-storage has been documented in the area encompassing the High Rainfall Province within the Yilgarn Craton [72]. The significant decline (a reduction of 14% in May–July was already observed for the years 1975–2004 when compared to 1900–1974) in autumn and early winter rainfall in the area since the 1970s [12], [13], [73], [74] has been accompanied by a shift from perennial to ephemeral streams, a regional decline in water-tables [72], a decline in the runoff coefficient, and the development of a new hydraulic regime [75]. Recent canopy death has been observed in the overstorey in this area [68], [76], generally on shallow soils around granite outcrops. This may be contradictory to the suggestion that on-flow sites at the base of granite outcrops may serve as refugia for taller vegetation in lower rainfall areas. However, on-flow areas with deep soils below granite outcrops will have greatest access to moisture via rainfall and runoff as the regolith continues to dry. The open forest of this area currently accesses moisture from deep, highly weathered lateritic soil profiles that store a large proportion of winter rains [77], [78]. As the water table further declines, forests on the shallowest soils of the region that have low water holding capacity, will be first affected [67], [68]. The hydrology of the area may be completely transformed when the regolith dries and groundwater becomes disconnected from the stream zone [73] and water gaining on-flow sites become increasingly important for biodiversity.

### The influence of disturbance history

In mediterranean-climate ecosystems fire regimes have a considerable influence on vegetation structure and also composition [63], [79]. Thus, where fire is sufficiently frequent it can determine species composition [79] and may become a major driver of vegetation structural change under climate change [80], [81]. The sites included in this study have an array of disturbance histories that may affect understorey vegetation structure. This should not be expected to greatly influence PVF structure in tall forest, where overstorey height and cover provides a distinct signature. However, in areas of high intensity fire (e.g. Mt Cooke, January 2003, 8 years prior to imagery being flown) or with low vegetation height and frequent fires e.g. Chiddarcooping [82], [83], fire may have a significant influence on PVF that may be age-since-fire specific, or related to particular fire regime history. Recognition that disturbance in these fire-prone landscapes may temporarily (or permanently under regime shifts) change vegetation structure [62], [63], [79] must be accommodated in vegetation mapping [84].

## Conclusion

Our study demonstrates the utility of enabling technologies such as LiDAR for identifying and mapping putative climate change refugia. Using granite outcrops in the SWAFR as a case study we found that the vegetation around outcrops included a wide range of structural classes, reflecting differences in local topography, soil depth and water influx associated with the large diversity of habitats found there [31], [85]. Under a rainfall reduction scenario predicted for the region, we were able to identify areas where vegetation structure may be likely to persist for longest, therefore providing safe havens for the biota under climate change. However, we acknowledge that interactions such as fire and declining water tables also influence response to climate change [76], [79]–[81], [86]. In addition, our projections are likely to be conservative since current vegetation structure may not yet reflect the major changes in rainfall reduction experienced in the years 2000–2010.

## References

[1] Parmesan C, Yohe G (2003) A globally coherent fingerprint of climate change impacts across natural systems. Nature 421: 37–42. 12511946 10.1038/nature01286

[2] Penuelas J, Boada M (2003) A global change-induced biome shift in the Montseny mountains (NE Spain). Glob Change Biol 9: 131–140.

[3] Rosenzweig C, Karoly D, Vicarelli M, Neofotis P, Wu Q, et al (2008) Attributing physical and biological impacts to anthropogenic climate change. Nature 453: 353–357. 18480817 10.1038/nature06937

[4] Keppel G, Van Niel KP, Wardell-Johnson GW, Yates CJ, Byrne M, et al (2011) Refugia: identifying and understanding safe havens for biodiversity under climate change. Global Ecol Biogeogr 21: 393–404.

[5] Loarie SR, Carter BE, Hayhoe K, McMahon S, Moe R, et al (2008) Climate change and the future of California's endemic flora. PloS One 3: e2502. 18648541 10.1371/journal.pone.0002502 PMC2481286

[6] Klein C, Wilson K, Watts M, Stein J, Berry S, et al (2009) Incorporating ecological and evolutionary processes into continental-scale conservation planning. Ecol Appl 19: 206–217. 19323184 10.1890/07-1684.1

[7] Game ET, Lipsett-Moore G, Saxon E, Peterson N, Sheppard S (2011) Incorporating climate change adaptation into national conservation assessments. Glob Change Biol 17: 3150–3160.

[8] Franklin J, Davis FW, Ikegami M, Syphard AD, Flint LE, et al (2013) Modeling plant species distributions under future climates: how fine scale do climate projections need to be? Glob Change Biol 19: 473–483. 10.1111/gcb.12051 23504785

[9] Austin MP, Van Niel KP (2011) Improving species distribution models for climate change studies: variable selection and scale. J Biogeogr 38: 1–8.

[10] Ford KR, Ettinger AK, Lundquist JD, Raleigh MS, Lambers JHR (2013) Spatial Heterogeneity in Ecologically Important Climate Variables at Coarse and Fine Scales in a High-Snow Mountain Landscape. PloS One 8: e65008. 23762277 10.1371/journal.pone.0065008 PMC3676384

[11] Hopper SD, Gioia P (2004) The Southwest Australian Floristic Region: Evolution and conservation of a global hot spot of biodiversity. Annu Rev Ecol Syst 35: 623–650.

[12] Bates BC, Hope P, Ryan B, Smith I, Charles S (2008) Key findings from the Indian Ocean Climate Initiative and their impact on policy development in Australia. Clim Change 89: 339–354.

[13] CSIRO (2007) Climate Change in Australia. Technical report 2007. Melbourne: CSIRO.

[14] Fitzpatrick MC, Gove AD, Sanders NJ, Dunn RR (2008) Climate change, plant migration, and range collapse in a global biodiversity hotspot: the Banksia (Proteaceae) of Western Australia. Glob Change Biol 14: 1337–1352.

[15] Yates CJ, McNeill A, Elith J, Midgley GF (2010) Assessing the impacts of climate change and land transformation on Banksia in the South West Australian Floristic Region. Divers Distrib 16: 187–201.

[16] Reside AE, VanDerWal J, Phillips B, Shoo LP, Rosauer DF, et al.. (2013) Climate change refugia for terrestrial biodiversity: Defining areas that promote species persistence and ecosystem resilience in the face of global climate change. Gold Coast, Australia: National Climate Change Adaptation Research Facility.

[17] Ashcroft MB, Gollan JR (2013) Moisture, thermal inertia, and the spatial distributions of near-surface soil and air temperatures: Understanding factors that promote microrefugia. Agr Forest Meteorol 176: 77–89.

[18] Wulder MA, Bater CW, Coops NC, White JC (2008) The role of LiDAR in sustainable forest management. Forest Chron 84: 1–20.

[19] Mallet C, Bretar F (2009) Full-waveform topographic lidar: State-of-the-art. ISPRS-J Photogramm Remote Sens 64: 1–16.

[20] Wulder MA, White JC, Nelson RF, Naesset E, Orka HO, et al (2012) Lidar sampling for large-area forest characterization: A review. Remote Sens Environ 121: 196–209.

[21] Simonson WD, Allen HD, Coomes DA (2013) Remotely sensed indicators of forest conservation status: Case study from a Natura 2000 site in southern Portugal. Ecol Indic 24: 636–647.

[22] Schut AGT, Wardell-Johnson G, Baran I. Canopy profiling for vegetation mapping in south-western Australian forested ecosystems. Available: http://www.isprs-ann-photogramm-remote-sens-spatial-inf-sci.net/I-7/365/2012/isprsannals-I-7-365-2012.pdf. Accessed 19 November 2013.

[23] Sun D, Hnatiuk RJ, Neldner VJ (1997) Review of vegetation classification and mapping systems undertaken by major forested land management agencies in Australia. Aust J Bot 45: 929–948.

[24] Sala OE, Chapin FS, Armesto JJ, Berlow E, Bloomfield J, et al (2000) Biodiversity - Global biodiversity scenarios for the year 2100. Science 287: 1770–1774. 10710299 10.1126/science.287.5459.1770

[25] Klausmeyer KR, Shaw MR (2009) Climate Change, Habitat Loss, Protected Areas and the Climate Adaptation Potential of Species in Mediterranean Ecosystems Worldwide. PloS One 4: e6392. 19641600 10.1371/journal.pone.0006392 PMC2712077

[26] Sander J, Wardell-Johnson GW (2012) Defining and characterizing high-rainfall Mediterranean climates. Plant Biosyst 146: 1–460.

[27] Cowling RM, Rundel PW, Lamont BB, Arroyo MK, Arianoutsou M (1996) Plant diversity in mediterranean-climate regions. Trends Ecol Evol 11: 362–366. 21237880 10.1016/0169-5347(96)10044-6

[28] Myers N, Mittermeier RA, Mittermeier CG, da Fonseca GAB, Kent J (2000) Biodiversity hotspots for conservation priorities. Nature 403: 853–858. 10706275 10.1038/35002501

[29] CSIRO BOM (2007) Climate Change in Australia: Observed Changes and Projections. Melbourne: CSIRO and the Australian Bureau of Meteorology.

[30] Twidale CR (1997) The great age of some Australian landforms: examples of, and possible explanations for, landscape longevity. Geology Society, London, Special Publications 120: 13–23.

[31] Hopper SD, Brown AP, Marchant NG (1997) Plants of Western Australian granite outcrops. J Roy Soc WA 80: 141–158.

[32] Mignaut T, Senterre B, Muller JV, Lejoly J, Parmentier I (2010) Shrubby and forest fringe communities of the inselberg-rainforest ecotone in Atlantic Central Africa. Plant Ecol Evol 143: 128–137.

[33] Withers PC, Edward DH (1997) Terrestrial fauna of granite outcrops in Western Australia. J Roy Soc WA 80: 159–166.

[34] Pinder AM, Halse SA, Shiel RJ, McRae JM (2000) Granite outcrop pools in south-western Australia: foci of diversification and refugia for aquatic invertebrates. J Roy Soc WA 83: 149–161.

[35] Burke A (2003) Inselbergs in a changing world - global trends. Divers Distrib 9: 375–383.

[36] Michael DR, Lindenmayer DB, Cunningham RB (2011) Managing rock outcrops to improve biodiversity conservation in Australian agricultural landscapes. Ecol Manag Rest 11: 43–50.

[37] Fernie N (1930) Water supplies from rock catchments in the Western Australian Wheatbelt. J Inst Engineers 2: 198–208.

[38] Bettenay E, Hingston FJ (1963) The quality of groundwaters in the Central Wheatbelt of W.A. J Dep Agr WA 4: 216–220.

[39] Burke A (2002) Properties of soil pockets on arid Nama Karoo inselbergs - the effect of geology and derived landforms. J Arid Environ 50: 219–234.

[40] Verboom WH, Pate JS (2003) Relationships between cluster root-bearing taxa and laterite across landscapes in southwest Western Australia: an approach using airborne radiometric and digital elevation models. Plant Soil 248: 321–333.

[41] Orians GH, Milewski AV (2007) Ecology of Australia: the effects of nutrient-poor soils and intense fires. Biol Rev Camb Philos Soc 82: 393–423. 17624961 10.1111/j.1469-185X.2007.00017.x

[42] Hopper SD (2009) OCBIL theory: towards an integrated understanding of the evolution, ecology and conservation of biodiversity on old, climatically buffered, infertile landscapes. Plant Soil 322: 49–86.

[43] Lambers H, Brundrett MC, Raven JA, Hopper SD (2010) Plant mineral nutrition in ancient landscapes: high plant species diversity on infertile soils is linked to functional diversity for nutritional strategies. Plant Soil 334: 11–31.

[44] Miura N, Jones SD (2010) Characterizing forest ecological structure using pulse types and heights of airborne laser scanning. Remote Sens Environ 114: 1069–1076.

[45] Goetz S, Steinberg D, Dubayah R, Blair B (2007) Laser remote sensing of canopy habitat heterogeneity as a predictor of bird species richness in an eastern temperate forest, USA. Remote Sens Environ 108: 254–263.

[46] Schimper AFW (1903) Plant geography on a physiological basis. Oxford: Clarendon Press.

[47] Specht RL (1970) Vegetation. In: Leeper GW, editor. The Australian environment. Melbourne: CSIRO and Melbourne University Press. pp. 44–67.

[48] Beard JS (1990) Plant life of Western Australia. Kenthurst: Kangaroo Press.

[49] Rundel PW (2001) Mediterranean-climate ecosystems. In: Levis S, editor. Encyclopedia of Biodiversity. New York: Academic Press. pp. 145–159.

[50] Wardell-Johnson G, Williams M (1996) A floristic survey of the Tingle Mosaic, south-western Australia: applications in land use planning and management. J Roy Soc WA 79: 249–276.

[51] Jaskierniak D, Lane PNJ, Robinson A, Lucieer A (2011) Extracting LiDAR indices to characterise multilayered forest structure using mixture distribution functions. Remote Sens Environ 115: 573–585.

[52] Reitberger J, Schnorr C, Krzystek P, Stilla U (2009) 3D segmentation of single trees exploiting full waveform LIDAR data. ISPRS-J Photogramm Remote Sens 64: 561–574.

[53] Morsdorf F, Marell A, Koetz B, Cassagne N, Pimont F, et al (2010) Discrimination of vegetation strata in a multi-layered Mediterranean forest ecosystem using height and intensity information derived from airborne laser scanning. Remote Sens Environ 114: 1403–1415.

[54] Richards JA, Jia X (1999) Remote sensing digital image analysis : an introduction. Berlin; New York: Springer.

[55] Hijmans RJ, Cameron SE, Parra JL, Jones PG, Jarvis A (2005) Very high resolution interpolated climate surfaces for global land areas. Int J Climatol 25: 1965–1978.

[56] Golbraikh A, Tropsha A (2002) Beware of q(2)!. J Mol Graphics Modell 20: 269–276. 10.1016/s1093-3263(01)00123-1 11858635

[57] Pearce K, Holper P, Mandy, Hopkins M, Bouma W, et al. (2007) Climate Change in Australia. Technical Report 2007, page 71. http://www.climatechangeinaustralia.gov.au/: Accessed 11 November 2013.

[58] Pate JS, Beard JS (1984) Kwongan, Plant Life of the Sandplain: Biology of a South-West Australian Shrubland Ecosystem. Nedlands: University of Western Australia Press.

[59] Keppel G, Wardell-Johnson GW (2012) Refugia: keys to climate change management. Glob Change Biol 18: 2389–2391.

[60] Bergen KM, Goetz SJ, Dubayah RO, Henebry GM, Hunsaker CT, et al (2009) Remote sensing of vegetation 3-D structure for biodiversity and habitat: Review and implications for lidar and radar spaceborne missions. J Geohys Res, G 114: 1–13.

[61] Korhonen L, Korpela I, Heiskanen J, Maltamo M (2011) Airborne discrete-return LIDAR data in the estimation of vertical canopy cover, angular canopy closure and leaf area index. Remote Sens Environ 115: 1065–1080.

[62] Hopkins AJM, Robinson CJ (1981) Fire induced structural change in a Western Australian Woodland. Aust J Ecol 6: 177–188.

[63] Burrows ND (2013) Fire dependency of a rock-outcrop plant Calothamnus rupestris (Myrtaceae) and implications for managing fire in south-western Australian forests. Aust J Bot 61: 81–88.

[64] Bindon PR (1997) Aboriginal people and granite domes. J Roy Soc WA 80: 173–179.

[65] Laing IAF, Hauck EJ (1997) Water harvesting from granite outcrops in Western Australia. J Roy Soc WA 80: 181–184.

[66] Poot P, Hopper SD, Van Diggelen JMH (2012) Exploring rock fissures: does a specialized root morphology explain endemism on granite outcrops? Ann Bot 110: 291–300. 22238122 10.1093/aob/mcr322 PMC3394634

[67] Poot P, Veneklaas EJ (2013) Species distribution and crown decline are associated with contrasting water relations in four common sympatric eucalypt species in southwestern Australia. Plant Soil 364: 409–423.

[68] Matusick G, Ruthrof K, Brouwers N, Dell B, Hardy GJ (2013) Sudden forest canopy collapse corresponding with extreme drought and heat in a mediterranean-type eucalypt forest in southwestern Australia. Eur J Forest Res 132: 1–14.

[69] Brouwers N, Matusick G, Ruthrof K, Lyons T, Hardy G (2013) Landscape-scale assessment of tree crown dieback following extreme drought and heat in a Mediterranean eucalypt forest ecosystem. Landsc Ecol 28: 69–80.

[70] Simonson WD, Allen HD, Coomes DA (2012) Use of an Airborne Lidar System to Model Plant Species Composition and Diversity of Mediterranean Oak Forests. Conserv Biol 26: 840–850. 22731687 10.1111/j.1523-1739.2012.01869.x

[71] Ashcroft MB, Gollan JR, Warton DI, Ramp D (2012) A novel approach to quantify and locate potential microrefugia using topoclimate, climate stability, and isolation from the matrix. Glob Change Biol 18: 1866–1879.

[72] Hughes JD, Petrone KC, Silberstein RP (2012) Drought, groundwater storage and stream flow decline in southwestern Australia. Geophys Res Lett 39: L03408.

[73] Reed AJ, Barrett KL, Croton JT (2012) Future streamflows from the northern jarrah forest: Learnings from the Wungong Catchment Trial. Perth: Water Corporation.

[74] Croton JT, Boniecka LH, Ruprecht J, Bari M (2005) Salinity and Land Use Impacts Series. Perth: Department of Environment.

[75] Petrone KC, Hughes JD, Van Niel TG, Silberstein RP (2010) Streamflow decline in southwestern Australia, 1950–2008. Geophys Res Lett 37.

[76] Wardell-Johnson GW, Keppel G, Sander J (2011) Climate change impacts on the terrestrial biodiversity and carbon stocks of Oceania. Pacific Conserv Biol 17: 220–240.

[77] Schofield NJ, Stoneman GL, Loh IC (1989) Hydrology of the Jarrah forest. In: Dell B, Havel JJ, Malajczuk N, editors. The Jarrah forest: a complex mediterranean ecosystem. Dordrecht: Kluwer Academic Publishers. pp. 179–201.

[78] Macfarlane C, Bond C, White DA, Grigg AH, Ogden GN, et al (2010) Transpiration and hydraulic traits of old and regrowth eucalypt forest in southwestern Australia. Forest Ecol Manag 260: 96–105.

[79] Bond WJ, Woodward FI, Midgley GF (2005) The global distribution of ecosystems in a world without fire. New Phytol 165: 525–537. 15720663 10.1111/j.1469-8137.2004.01252.x

[80] Bradstock RA (2010) A biogeographic model of fire regimes in Australia: current and future implications. Global Ecol Biogeogr 19: 145–158.

[81] Mucina L, Wardell-Johnson GW (2011) Landscape age and soil fertility, climatic stability, and fire regime predictability: beyond the OCBIL framework. Plant Soil 341: 1–23.

[82] Hopper SD (2000) Creation of conservation and managing fire on granite outcrops - A case study of Chiddarcooping Nature Reserve in the Western Australian wheatbelt. J Roy Soc WA 83: 173–186.

[83] Yates CJ, Hopper SD, Brown A, Van Leeuwen S (2003) Impact of two wildfires on endemic granite outcrop vegetation in Western Australia. J Veg Sci 14: 185–194.

[84] Mucina L, Rutherford MC (2006) The vegetation of South Africa, Lesotho and Swaziland. Pretoria, South Africa: South African National Biodiversity Institute.

[85] Porembski S, Seine R, Barthlott W (1997) Inselberg vegetation and the biodiversity of granite outcrops. J Roy Soc WA 80: 193–199.

[86] Fried JS, Torn MS, Mills E (2004) The impact of climate change on wildfire severity: A regional forecast for northern California. Clim Change 64: 169–191.

